# No evidence of moral licensing in a laboratory bribe-taking task

**DOI:** 10.1038/s41598-022-16800-4

**Published:** 2022-08-16

**Authors:** Štěpán Bahník, Marek Vranka

**Affiliations:** Faculty of Business Administration, Prague University of Economics and Business, náměstí Winstona Churchilla 4, Prague, 130 67 Czech Republic

**Keywords:** Human behaviour, Psychology

## Abstract

Moral licensing posits that previous moral acts increase the probability of behaving immorally in the future. According to this perspective, rejecting bribes, even because they are too small, would create a kind of “license” for taking (presumably larger) bribes in the future. On the other hand, the desire for consistency in behavior predicts that previous rejection of bribes will increase the probability of rejection for bribes offered in the future. Using a laboratory task modeling the decision to take a bribe, we examined how resisting and succumbing to the temptation to take a bribe affects later bribe-taking. Participants (*N* = 297) were offered either low bribes first and high bribes later or vice versa. Low bribes were in general rejected more often and the results showed some weak, nonsignificant evidence that bribe-taking may be influenced by the order of the sizes of offered bribes. However, there was no evidence of an increased probability of taking bribes after being offered the low bribes first and thus no evidence in support of the moral licensing effect.

## Introduction

Many studies have recently started experimentally exploring corruption, that is bribe-giving as well as bribe-taking, in laboratory conditions, usually using simple one-shot games (for a review, see^1^). However, the opportunity to act corruptly often presents itself repeatedly in the world outside of a laboratory. In the present study, we therefore explore how succumbing or resisting temptation to take a bribe affects behavior when the opportunity to take a bribe arises again.

According to the standard economic model usually applied for the analysis of criminal behavior^2,3^, self-interested individuals should opt for dishonest acts whenever the difference between the expected benefits from dishonesty and the expected costs associated with punishment is higher than the benefits from honest activities^3^. Yet, in reality, people often behave honestly even when the probability of punishment is negligible and thus the expected costs of being dishonest seem to be practically zero^4,5^. This discrepancy can be explained by the existence of internal psychological costs of dishonest behavior, which may be a consequence of internalized social norms and hesitation to harm others^6,7^.

Even though people seem to intuitively refrain from directly harming specific others^8^, when victims of their actions are more abstract, distant, and perceived less as specific individuals, the evaluation of the psychological costs of wrong-doing becomes less straightforward. For example, the anticipated internal costs of acting dishonestly may then depend on accidental aspects of a given situation, such as how salient is currently morality^9^ (but see^10^), or one’s self-concept^6^. Moreover, people can retain a positive self-view by attenuating perceived immorality of the behavior in question^11,12^, especially when there is enough time for deliberation^13^. Therefore, people will more likely act dishonestly when self-justification of dishonest behavior is easier^14–16^, for example because the harm seems to be small^6,17^, or because the responsibility for the dishonest act can be diffused^18^ or delegated to someone else^19^.

When the opportunity to act dishonestly is repeated, people’s previous behavior may affect the current internal costs of dishonesty. According to the theory behind the so-called moral licensing effect^20^, behaving morally increases the probability of subsequent dishonest acts, because previous positive acts protect against appearing and feeling bad when one does something immoral. For example, writing about oneself using morally positive trait words (e.g., kind, generous) decreased the amount subsequently donated to a charity^21^. In other studies, even only imagined moral acts^22^ or expressed intentions to do something good^23^ led to lower generosity towards a charity and decreased intentions to act selflessly in the future. From the perspective of the moral self-regulation framework, the positive self-perception activated in one’s mind by moral acts helps to perceive oneself as moral even when doing something wrong and it also decreases additional self-image gains from subsequent moral deeds^21^.

On the other hand, many social psychological theories posit that people strive for consistency and thus previous actions may increase the probability of behaving in a similar fashion^24^. After a moral deed, one may perceive oneself as someone “who behaves like this in the given circumstances” and thus repeat similar behavior in the future. In case when one does something immoral, he or she may automatically attempt to justify or rationalize such behavior and thanks to this moral disengagement^11^ be more ready to repeat the act in the future.

In the present study, we explore which of these theoretical perspectives is in line with bribe-taking behavior in a laboratory setting. We opt for a design in which the opportunity to take a bribe is offered repeatedly during a task that participants are asked to perform according to given rules^25^. When offered a bribe, participants can decide whether to break the rules to get an additional reward while simultaneously hurting a third party, or uphold the rules, ignoring the bribe. Based on the results of previous studies^25,26^, we expect that participants will be less likely to take a bribe and cause harm to the third party when the size of the offered bribe is low. Therefore, those offered low bribes will be on average more likely to reject them than those offered higher bribes. At the same time, rejecting an offered bribe is likely to be perceived as moral^25^. However, it is not clear how participants will respond to a sudden change in sizes of the offered bribes. In line with the preference for consistent behavior^24^, it is possible that resisting initial lower bribes will lead them to continue to refuse bribes even when their size increases. That is, their honest behavior may become routine and automatic. On the other hand, not taking initial low bribes may provide a “moral license” to act more dishonestly later and take a higher number of the higher bribes^20^. Similarly, those who encountered higher bribes first may become morally disengaged^11^ and lose any inhibition for accepting them and thus be more likely to accept even the lower bribes offered later. Alternatively, they may find the lower bribes relatively less attractive when compared to higher bribes they have encountered previously and thus be more likely to reject the lower bribes. To disentangle the alternative explanations and avoid the so-called “donut design” commonly found in moral licensing studies, we also used a control group which was given bribes of all sizes throughout the task.

## Methods

Preregistration of the study as well as data, analysis scripts, and materials can be found at https://osf.io/nwdx8/. The study was performed in accordance with the principles of the Declaration of Helsinki and all relevant regulations for conducting psychological studies in the Czech Republic. Ethical approval was waived by the local Research Ethics Committee at the Center of science and research at the Faculty of Business Administration at the Prague University of Economics and Business in view of the low-risk nature of the study’s design and anonymity of the collected data. All participants provided informed consent and were assured of anonymity and confidentiality of their responses.

### Participants

Participants were recruited from a laboratory subject pool for an on-line study. The participant pool consists mostly of university students (~ 74%) and women (~ 71%). For the sake of higher anonymity, we did not ask the participants of the present studies any demographic questions. We sent 1880 invitations; 332 participants started the study, 308 of them started the task itself, and 302 finished the whole study. One participant with missing data from 3 or more trials (for example due to internet connection failure) was excluded from analysis according to a pre-registered exclusion criterion. To exclude participants who did not respond or responded randomly, we excluded 4 participants who did not sort at least 10 times the object according to either its color or shape. We performed the analysis with the remaining 297 participants. The participants who finished the study took on average 11.5 min to complete it and earned on average 155 CZK (~ 6.8 USD) for themselves. The experiment had sufficient power (0.80) to detect an effect *d* = 0.40, assuming a comparison of two groups, each with one third of the participants. While meta-analyses of the moral licensing effects suggest smaller effect sizes (*d* = 0.31^34^), unlike a lot of studies on the topic, we used repeated measurement for the target behavior and repeated behavior for induction of the licensing, which led us to expect a larger studied effect size in the present study.

### Procedure

**Figure 1:**
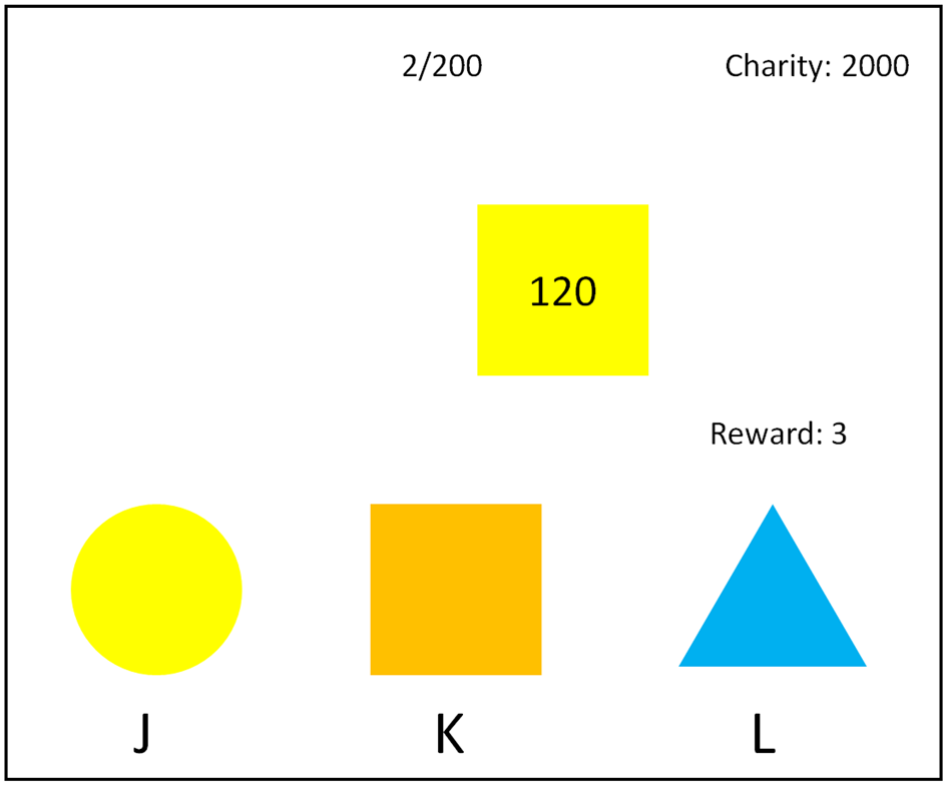
An illustration of a computer screen seen by a participant. The top row shows information about the number of the current trial, the total number of trials, and the number of points currently assigned to the charity organization. In the middle of the screen, an object (a yellow square in this case) is moving from the left side of the screen to the right. The current participant’s reward in points is shown to the right of the screen. In the bottom row, a participant sees which shapes and colors are assigned to keys “J,” “K,” and “L” in this trial (in this example, J is a yellow circle, K is an orange square, and L is a blue triangle). If the participant presses “J,” the object would be sorted by its color, that is correctly, and the participant would gain 3 points. If the participant presses “K,” the object would be matched to a wrong color, and would cause a loss of 200 points for the charity, but it would be sorted according to its shape, gaining the participant the 120 points marked on the object in addition to the 3 points awarded for each sorted object. Adapted from "Bureaucracy game: A new computer task for the experimental study of corruption," by M. A. Vranka and Š. Bahník, 2018, *Frontiers in Psychology, 9:1511*, p. 3. Copyright 2018 by Vranka and Bahník.

The experiment used a task simulating routine administrative work during which the worker is sometimes given an opportunity to take a bribe^25–28^. Participants were told to sort objects moving across a computer screen according to their color. The speed of the objects was calibrated to window size, so that they stayed on the screen for the same duration for all participants. The objects had three possible shapes (triangle, square, and circle) and colors (yellow, blue, and orange), both of which were determined randomly on each trial. The sorting was done by pressing one of three keys (“J”, “K”, and “L”), each of which was associated with a single color and shape (see Fig. 1 for an illustration of the task). Colors associated with the three keys were randomly determined for each trial and they were displayed prominently at the bottom of the screen. At the beginning of the task, 2000 points (corresponding to 200 CZK, ~ 8.7 USD) were allotted to a charity chosen by the participant before the task from two well-known Czech charitable organizations. The points participants earned during the task were converted to a monetary reward using the conversion rate 10 points = 1 CZK. If a key response led to an assignment to a wrong color, the charity lost 200 points. The loss simulated negative societal effects of not performing given work according to the given rule. Regardless of whether the object was sorted according to the rule or not, participants got a fixed reward of 3 points for each sorted object, which represented the salary given to a worker for performing their job. In any case the intended meaning of the incentives was not revealed to the participants. Finally, in trials where the two sorting criteria were mismatched, there was a 22.5% probability that a given object was associated with a “bribe”—which can also represent embezzled money or any gain from dishonest behavior in general. These objects were shown with a number corresponding to the value of the bribe, which a participant got if they sorted the object according to its shape instead of its color. The bribe sizes randomly varied from 30 to 180 points in 30-point increments. Each participant went through 200 trials of the task; that is 200 objects to sort.

The experiment was conducted online using a custom-written web application. Participants were explained the task, completed 10 practice trials and then proceeded with the task itself. The practice trials did not involve any reward or bribes and served only to accustom participants with the sorting task. Afterwards, participants were explained the possibility to earn additional money for themselves by breaking the sorting rule and sorting objects with numbers according to their shape instead of color. However, they were not told the size or probability of bribes.

### Design

Participants were randomly assigned to one of three conditions: in the control condition, bribes varied from 30 to 180 in all 200 trials. For the low–high condition, bribes varied from 30 to 90 in the first 100 trials and from 120 to 180 in the remaining 100 trials. In the high-low condition, the order was reversed. On each trial with a bribe, its size was determined randomly from the possible set of values for the condition and trial number.

## Results

Trial-level analysis was conducted using a mixed-effect linear regression using R packages lme4^29^ and lmerTest^30^. The incorrectness of object classification, i.e., taking a bribe, served as the dependent variable. The trials incorrectly sorted according to both shape and color as well as trials without a bribe were excluded. Order of the trial and a squared order of the trial (centered and rescaled to range from − 0.5 to 0.5) were included as covariates. The three conditions were compared using simple coding, where both low–high and high-low conditions were compared to the control condition. The model also included a bribe group coded as -0.5 for bribes 30–90 and 0.5 for bribes 120–180 and its interaction with the condition. Bribe size was included as a covariate using linear and quadratic contrasts with the ordering 30/120, 60/150, and 90/180. The interaction of bribe size and bribe group was also included in the model. Random intercepts for participants were included. Random slopes for participants were included for bribe size and bribe group to take into account that the effects of bribe size and bribe group can differ between participants.

**Figure 2:**
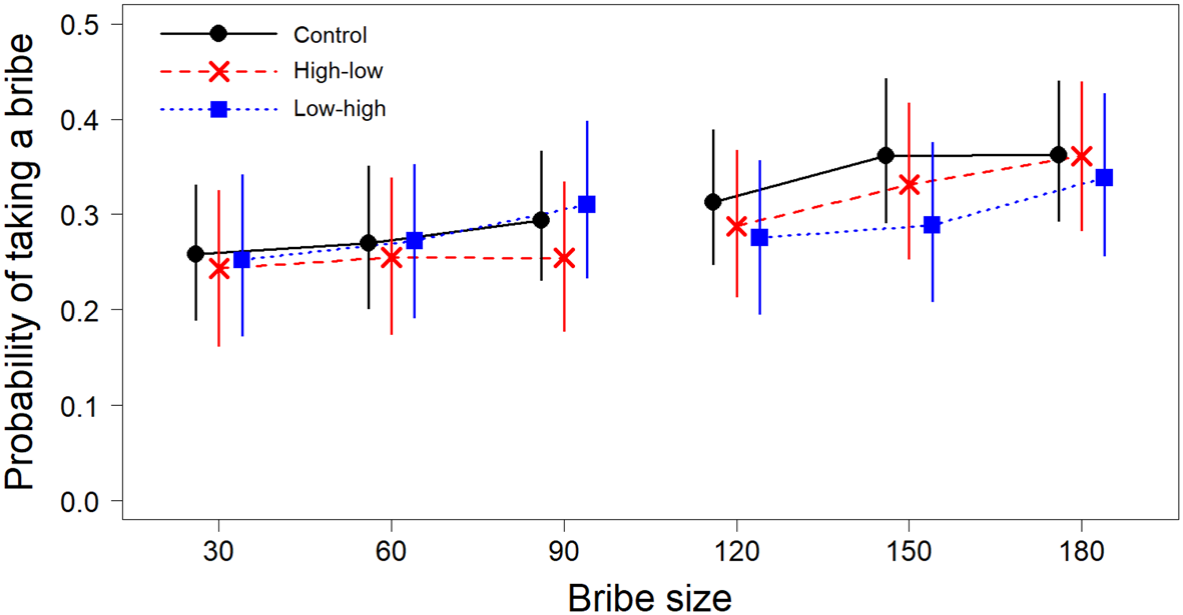
The predicted probability of taking a bribe based on a condition and bribe size. Unlike the model reported in the text, the model used in the figure did not include order effects, but included triple interaction between bribe size, bribe group, and condition. The main results were robust to the model specification, but the displayed model better recreates the observed averages. The error bars show 95% prediction intervals. The predictions and confidence intervals are based on bootstrapped estimates. Note that the displayed points are slightly shifted on the x-axis to not overlap.

Figure 2 shows the probabilities of taking bribes of different sizes in each experimental condition. Table 1 shows the results of the model. Neither the high-low group nor the low–high group differed in their overall rate of bribe-taking from the control group. Participants were more likely to take high bribes than low bribes and within each bribe group the rate of bribes taken also increased with bribe size. There was no quadratic effect of bribe size. The effect of bribe size differed between high and low bribes, suggesting that participants were more sensitive to the size of the bribe for high bribes, even though the effect of bribe size was significantly positive for both high and low bribes. There was no interaction between the bribe group and the quadratic effect of bribe size. Participants were somewhat less likely to take bribes in later trials. The effect of the squared trial order suggested that the decrease of the bribes taken was more pronounced in later trials.

**Table 1:** The results of Study 1.

	Bribe-taking
High-low (vs. Control) condition	−0.021
	(−0.124, 0.081)
Low–high (vs. Control) condition	−0.012
	(−0.116, 0.092)
Bribe group	0.057***
	(0.035, 0.078)
Bribe size (linear)	0.034***
	(0.023, 0.045)
Bribe size (quadratic)	−0.001
	(−0.010, 0.008)
Trial number (linear)	−0.027*
	(−0.051, −0.002)
Trial number (quadratic)	−0.020*
	(−0.037, −0.003)
High-low condition x Bribe group	−0.008
	(−0.057, 0.041)
Low–high condition x Bribe group	−0.045
	(−0.095, 0.005)
Bribe group x Bribe size (linear)	0.019*
	(0.001, 0.037)
Bribe group x Bribe size (quadratic)	−0.007
	(−0.025, 0.011)
Constant	0.293***
	(0.250, 0.336)
Observations	8,951

The numbers in parentheses represent 95% confidence intervals around the regression coefficients. Random effects are not shown for simplicity. *p < 0.05, **p < .0.01, ***p < 0.001.

Most importantly, the difference between taking high and low bribes did not differ between high-low and control conditions. However, the difference between high and low bribes was somewhat smaller for the low–high condition than for the control condition, even though the effect was not significant, *p* = 0.080. That is, while participants were more likely to take high bribes than low bribes in the control condition, *t*(106.6) = 4.37, *p* < 0.001, *b* = 0.071, 95% CI [0.039, 0.103], the effect of the bribe group was smaller and not significant in the low–high condition, *t*(87.4) = 1.24, *p* = 0.217, *b* = 0.022, 95% CI [−0.013, 0.056]. If the moral licensing effect was present, we would expect the difference in the opposite direction: that is, the effect of bribe group would be larger in the low–high condition than in the control condition given that participants in the low–high group would be more likely to take the high bribes due to moral licensing.

Other approaches toward the analysis were possible. The reported pre-registered analysis model did not include random slopes for the interaction between bribe size and bribe group, which Barr^31^ recommends including. Unlike in the reported model, the interaction between bribe group and linear contrast for bribe size was not significant in the model with random slopes for the interaction, *t*(307.9) = 1.64, *p* = 0.101, *b* = 0.019, 95% CI [− 0.004, 0.042]. However, we did not report results of this more complex model because it resulted in a singular fit, suggesting overfitting. Subsequent simplification of the model by removal of random effects and their correlations suggested by Bates et al.^32^ still resulted in a model with a singular fit or in models with worse Akaike information criterion than the pre-registered model, which we ultimately reported here for this reason. As the dependent variable is binary, it would have been also possible to use a logistic mixed-effect regression. We used a linear regression rather than logistic regression based on the recommendation by Gomila^33^, as we are primarily interested in interactions that are harder to interpret in logistic models. Nevertheless, the results of the model with interaction random slopes and of the logistic regression model can be found on: https://osf.io/wzpdx/.

In an exploratory analysis, we looked at the effect of a previous bribe-taking decision on bribe-taking to examine whether the licensing effect might occur on a trial-by-trial basis. Specifically, we used only data from the control condition where the bribes were of the full range of sizes throughout the task. We conducted a mixed-effect linear regression with the incorrectness of object classification as the dependent variable. We included the linear effect of the bribe size (including all bribe sizes from 30 to 180), previous decision to take a bribe, and bribe size during the previous decision as predictors. We also included an interaction for the latter two variables in the model. We used random intercepts for participants and random slopes for all main effects of the predictor variables (inclusion of random slopes for the interaction resulted in a model with a singular fit, but similar results). All the variables were transformed to have a range of 1 and centered.

Participants were more likely to take a bribe when they took the previously offered bribe, *t*(62.0) = 2.98, *p* = 0.004, *b* = 0.078, 95% CI [0.027, 0.129], showing consistency in the bribe-taking behavior. The size of the previously offered bribe did not significantly affect bribe-taking, *t*(72.2) = −1.66, *p* = 0.102, *b* = −0.026, 95% CI [−0.057, 0.005]. However, the relationship between bribe-taking on two subsequent trials differed based on the size of the previously offered bribe, *t*(182.1) = −2.46, *p* = 0.015, *b* = −0.075, 95% CI [−0.135, −0.015]. That is, if participants took a bribe when it had been previously offered, they were less likely to take a subsequent bribe if the previously taken bribe was of higher size, *t*(24.8) = −1.98, *p* = 0.059, *b* = −0.069, 95% CI [−0.138, −0.001], even though the effect was not significant. This effect could be due to a motivation to achieve a specific total reward which would be nearer if the taken bribe was of a larger size. On the other hand, if participants did not take a bribe when it had been previously offered, the effect of the rejected bribe size was smaller, *t*(44.7) = −0.91, *p* = 0.370, *b* = −0.017, 95% CI [−0.055, 0.020].

Finally, as an additional exploratory analysis, we calculated the correlation of the proportion of bribes taken in the first and second half of all trials for the high-low group, *r*_S_ = 0.72, 95% CI [0.57, 0.82], *p* < 0.001, and the low–high group, *r*_S_ = 0.84, 95% CI [0.74, 0.90], *p* < 0.001. The difference between the correlation sizes was significant, *z* = −2.01, *p* = 0.045. The higher correlation in the low–high group suggests that behavior was more consistent across the two blocks in this condition. In the case of moral licensing, we would expect the correlation to be lower in the low–high group, as not taking the initial lower bribes would be associated with higher probability of taking subsequent higher bribes.

## Discussion

As in previous studies using the task^25,26^, participants were more likely to take higher bribes. However, the difference was somewhat smaller for the condition that was initially offered only low bribes and afterward only high bribes. This result goes against the prediction of moral licensing theory, according to which behaving honestly by resisting lower bribes increases subsequent bribe-taking. In line with the alternative perspective, it is possible that participants could have learned to reject the offered bribes and thus were more likely to refuse even more tempting bribes later in the task. This is supported by the stronger correlation between bribe taking in the first and second half of trials in the condition where lower bribes were offered first. Participants generally took fewer bribes in later trials, so it is also possible that this tendency contributed to the lack of a significant difference in the proportion of bribes taken between high and low bribes. Participants could have also had in mind an expected reward from the experiment which they might have achieved by taking the low bribes and they thus did not feel a need to take higher bribes later in the task. However, these two latter explanations would also predict a decrease in the proportion of taken low bribes in the condition that was offered first high bribes and later low bribes, which was not observed. Given that the difference between the control condition and the condition offered first low and later high bribes was not statistically significant and given that the result was also somewhat sensitive to specification of the regression model, it is also possible that the observed difference between the two conditions might have been a fluke. The difference in bribe-taking between low and high bribes was also smaller than in previous research using the task^25,26^, which means that the manipulation might have had a weaker impact, which could have contributed to the small obtained effects. A future study may attempt to replicate the effect and distinguish between its possible explanations, if the effect proves to be reliable.

According to the review of moderators^34^ of the licensing effect, licensing is more likely to be observed when individuals think concretely and do not connect their behavior to their underlying values. In our study, participants made concrete choices with tangible outcomes and there were no prompts to suggest a connection between their decisions and their values. Moreover, participants had the opportunity to resist temptation and gain moral credits repeatedly, instead only once as is common in other licensing studies. The initial behavior (i.e., resisting lower bribe) was also the same as the target behavior (i.e., resisting higher bribe). Our task therefore seems to be well suited for observing the licensing effect—in case it actually exists. However, it is possible that some features of the task could have influenced the lack of evidence for moral licensing. For example, the private nature of behavior in the task could have led people to focus on their preferences^35^ and behave more consistently. On the other hand, there is also some evidence that people are more likely to exhibit licensing behavior when the target act is private^36^. Furthermore, the task aims to model bribe-taking, but lacks social interaction which is an important feature of many types of corruption. Even though some results suggest validity of the task^25,26^, its external validity has never been directly tested.

The lack of support for the moral licensing effect found in our study is in line with a growing number of unsuccessful attempts to observe the effect across different domains^24,37–39^. Even though two meta-analytical studies^40,41^ concluded that there is evidence for the existence of the effect, more recent analysis by Kuper and Bott^42^ demonstrated that the available evidence in its favor may be inflated by publication bias and the true effect size might well be close to *d* = 0.

The results instead suggest that the tendency to behave honestly may be partly habitual. On the other hand, we did not find the opposite tendency of habitual cheating learned by acceptance of large bribes. In organizations, employees’ responsibility usually increases with seniority which—as the results suggest—might help reduce the overall rate of dishonest behavior. Initially, dishonest behavior has only a limited payoff and honesty thus becomes habitual even when responsibilities and thus payoff from dishonesty increase. Unlike in the present study, in the real world the size of the reward from dishonest behavior is often associated with the damage from the behavior to others. How the development of the size of benefits of dishonesty in time influences the rate of dishonest behavior in such situations is an open question for future research.

## Data Availability

The datasets generated and analyzed during the current study are available in the OSF repository, https://osf.io/nwdx8/.
